# Long COVID: From olfactory dysfunctions to viral Parkinsonism

**DOI:** 10.1002/wjo2.175

**Published:** 2024-04-08

**Authors:** Sanyukta Pandey, Vibha Bapat, Jancy Nixon Abraham, Nixon M. Abraham

**Affiliations:** ^1^ Department of Biology, Laboratory of Neural Circuits and Behaviour (LNCB) Indian Institute of Science Education and Research (IISER) Pune Maharashtra India; ^2^ Department of Life Sciences, Centre of Excellence in Epigenetics Shiv Nadar Institution of Eminence Gautam Buddha Nagar Uttar Pradesh India

**Keywords:** long COVID, neurodegeneration, neurotropism, olfactory dysfunctions, viral parkinsonism

## Abstract

Neurological and psychiatric complications continue to be a public health concern in long coronavirus disease 2019 (COVID‐19). This varies from olfactory dysfunctions such as parosmia to cognitive and emotional challenges. Historically, the surge of neurological disorders followed the viral pandemics, for example, the emergence of Encephalitis Lethargica after the outbreak of Spanish Influenza. During and after COVID‐19 infection, the problems associated with the sense of smell and the reports of affected olfactory and limbic brain areas are leading to a growing concern about the similarity with the symptoms and the pattern of degeneration observed at the onset of Parkinson's disease and Alzheimer's disease. These reports reveal the essentiality of long‐term studies of olfactory and cognitive functions in the post‐COVID era and the experiments using animal models to dissect the neural basis of these complications. In this manuscript, we summarize the research reporting the potential correlation between neurological disorders and viral pandemic outbreaks with a historical perspective. Further, we discuss the studies providing evidence of neurodegeneration due to severe acute respiratory syndrome coronavirus 2 infection by focusing on viral Parkinsonism.

## Key points

- Emergence of encephalitis lethargica and Parkinson's disease followed by Spanish Influenza.
- Neurological and psychiatric complications among coronavirus disease 2019 (COVID‐19) long‐haulers.
- Adverse effects of COVID‐19 infection on olfactory and limbic brain areas leading to plausible neurodegeneration.
- Conflicting evidence on neurotropism of severe acute respiratory syndrome coronavirus 2.
- Link between viral infections and the development of neurodegenerative diseases.
- Olfactory dysfunctions as a common factor between long COVID and Parkinson's Disease.

## INTRODUCTION

There has been an upsurge of neurological disorders followed by viral pandemics in history. The encephalitis lethargica (EL) that emerged during 1916–1917 closely followed the second wave of Spanish influenza.^1^, ^2^ While describing the symptoms of patients for the first time, Dr. Constantin von Economo classified the neurological complications into somnolent‐ophthamoplegic, hyperkinetic, and amyostatic‐akinetic.^1^, ^3^ Among these, amyostatic‐akinetic was the least common one and showed similarities with some of the Parkinson's‐like symptoms, which appeared much later, typically one to 5 years after the initial infection and other symptoms.^1^, ^4^ Incidentally, there were many reports of idiopathic Parkinson's disease (PD) in the general population 5–10 years after the pandemic. A multitude of cases were reported over the next 42 years, that is, from 1918 to 1950, following the Spanish influenza and Von Economo epidemics. However, only a few cases of PD had been identified in the 43 years before 1915, although the efficiency of diagnosis and reporting during that period needs to be considered.^4^, ^5^ Further, various neurological symptoms, including motor deficits, delirium, and coma, had also been linked, although to a lesser extent, with other influenza pandemics, for example, the 1968 Hong Kong flu caused by the H3N2 virus.^6^, ^7^ These observations imply a potential correlation between the viral pandemic and the upsurge in neurological disorders.

As we are recovering slowly from another global health disaster of the coronavirus disease 2019 (COVID‐19) pandemic, serious concerns about its long‐term neurological adverse effects are looming. Thus far, COVID‐19 has claimed 7 million lives worldwide (WHO data, January 2024) and unprecedentedly challenged the public health system to deal with respiratory, metabolic, cardiovascular, and neurological complications.^8^, ^9^ Among the neurological complications, olfactory dysfunctions were prevalent with the infection of early variants of COVID‐19.^10^, ^11^, ^12^ The infection of non‐neuronal cells of nasal epithelium, and the observed microvascular damage in the olfactory bulb (OB) could explain the symptoms of hyposmia, anosmia, and parosmia.^13^, ^14^ All of these findings point to a direct and severe infection of the olfactory pathway, and its long‐term neurological impact is yet to be fully understood. When we look at the commonalities between severe acute respiratory syndrome coronavirus 2 (SARS‐CoV‐2) and H1N1 viruses, both primarily affect the respiratory system and manifest neurological complications.^15^ However, detecting the presence of virus in the central nervous system (CNS) at the early stage of disease, and thereby correlating it with the progression of pathology, is challenging. Given the upsurge of PD that followed the Spanish Influenza pandemic and EL, careful attention needs to be paid to the possibility that similar issues may arise as a result of infections with the SARS‐CoV‐2 virus. There is clearly a need for long‐term human and animal model studies of COVID‐19.^16^

The olfactory system is vulnerable to pathogenic infections because of its location in the rostralmost part of the brain and proximity to the nasal epithelium, which opens directly to the external environment through the nares.^17^, ^18^ The orthonasal pathway is considered to be one of the most plausible viral entry routes to the CNS. Such entry can lead to infection of the glial cell population of various brain regions, thereby causing neuroinflammation and cell death within both neuronal and non‐neuronal populations.^18^, ^19^, ^20^, ^21^ Shared vasculature and lymphatics between nasal epithelium and anterior‐most brain region, the olfactory bulb (OB), the pre‐cortical area that is responsible for odor information processing, could act as a path of entry for pathogens directly to the brain without crossing the blood–brain barrier (BBB).^22^ The accumulation of airborne viruses entering through the nares can find a suitable place to thrive in the upper recesses between the nasal mucosa and cribriform plate without even infecting the nasal epithelium. This can lead to non‐obstructive olfactory loss, which could explain the huge population of COVID‐19 asymptomatic patients with olfactory deficits.^23^, ^24^, ^25^ Studies provide evidence for virus invasion through olfactory, ocular, trigeminal, and vagus nerves.^26^, ^27^, ^28^ Although the presence of viral particles in the CNS and the brain morphology aberrations have been confirmed by different reports, the neurotropism of SARS‐CoV‐2 is still a question of debate.^14^, ^29^ Thus far, several neurological complications have been reported in subjects who have recovered from COVID‐19.^30^, ^31^ However, the more severe long‐term implications of the SARS‐CoV‐2 infection, such as its potential role in triggering the development of neurodegenerative diseases need to be investigated.

## NEUROLOGICAL AND PSYCHIATRIC MANIFESTATIONS OF VIRAL EPIDEMICS/PANDEMICS: A HISTORICAL PERSPECTIVE

**Figure 1:**
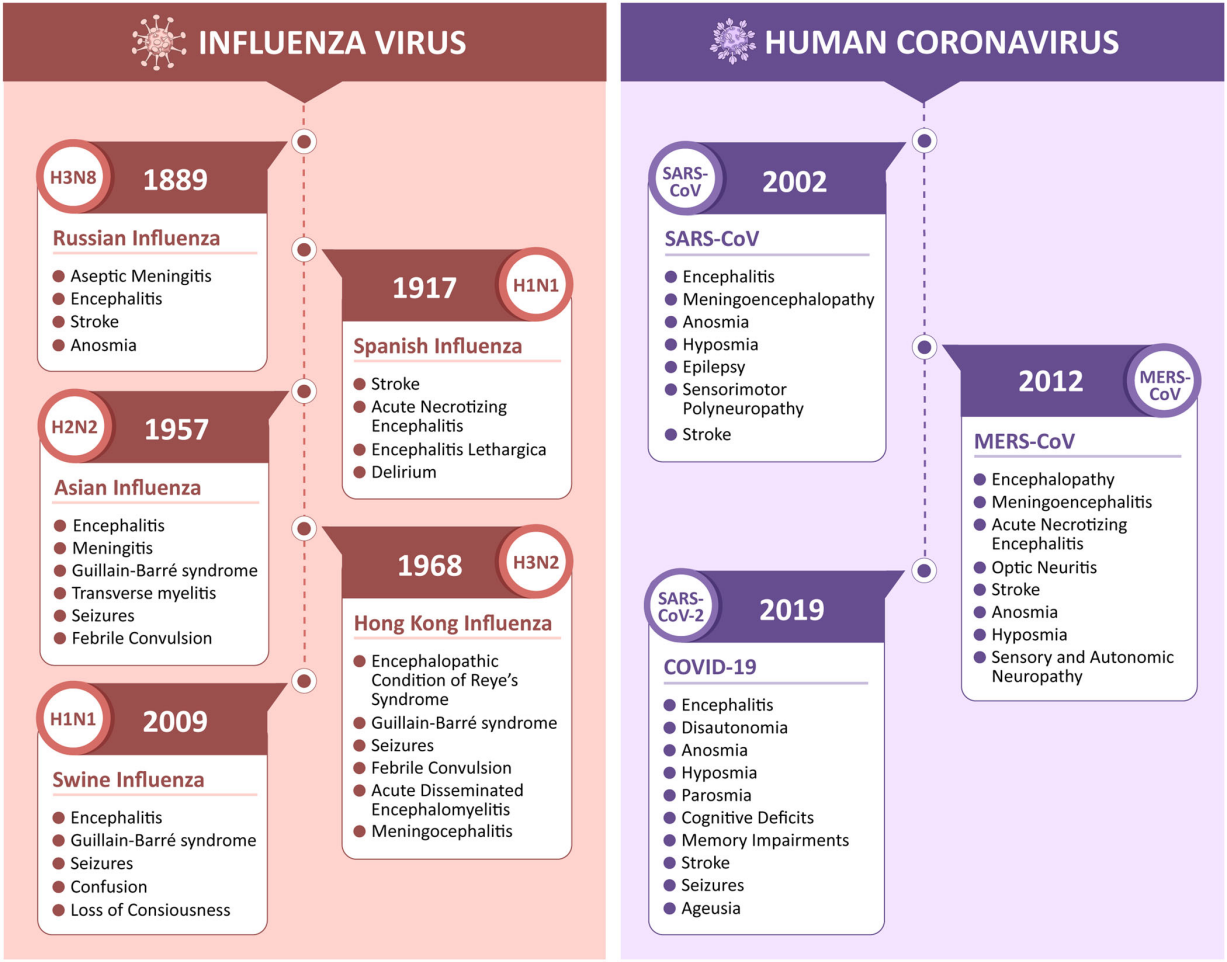
Timeline and neurological manifestations of major respiratory viral epidemics/pandemics. Influenza and human coronaviruses have been associated with a variety of neurological manifestations, including encephalitis, meningitis, stroke, Gullian‐Bar Syndrome, and seizures. Russian flu led to inflammatory central nervous system (CNS) complications and anosmia,^42^ Encephalitis Lethargica closely followed Spanish flu and presented severe CNS manifestations;^1^, ^43^ subsequently, Asian flu pandemics presented complications like encephalitis, meningitis, and convulsions.^41^, ^44^, ^45^ An upsurge in the H1N1 virus again in 2009‐10 led to what is infamously known as “Swine flu.”^46^ Human coronaviruses, including severe acute respiratory syndrome coronavirus (SARS‐CoV), Middle East respiratory syndrome (MERS), and SARS‐CoV‐2 are known to cause a multitude of CNS and peripheral nervous system (PNS) complications.^23^, ^47^

The “sweating sickness” that appeared in England in the 14th and 15th centuries could be the earliest recorded influenza epidemic.^32^, ^33^ During the late 15th century, Sydenham described an illness with whooping cough, violent motion in the lung, occasionally accompanied by vomiting and vertigo. This could be considered the first neurological manifestation of an influenza epidemic.^34^ The Russian flu pandemic that happened toward the end of the 19th century was characterized by the neurological sequelae of facial neuralgia, pneumogastric nerve neuritis, and occasional loss of smell (anosmia). Several psychiatric manifestations, such as despair and melancholy, also followed the Russian flu pandemic, coupled with suicidal thoughts, lethargy, and cataleptic episodes.^35^ Thereafter, the Spanish Influenza in 1917 was followed by a wave of Encephalitis Lethargica that affected more than one million people, as described by Constantin von Economo.^1^, ^36^, ^37^ Although it is unclear whether EL was caused by the influenza virus, the disease was characterized by problems associated with inflammation in the brain, including cerebrospinal meningitis and motor disorders.^38^, ^39^, ^40^ There were several similar neurological problems reported during the 1957 Influenza pandemic (Asian flu) as well. Encephalitis, visual abnormalities, ataxia, seizures, amyotrophy, and cognitive decline under Delirium were some of the common neurological problems observed during Influenza pandemics.^7^, ^41^ The neurological manifestations and the timeline of major viral epidemics/pandemics are shown in Figure 1.

An upsurge in PD cases was reported in the post‐EL period. Moreover, the reports of upper midbrain and substantia nigra damage in EL patients indicated a potential correlation between parkinsonism and EL. The presence of neurofibrillary tangles further confirmed the neurodegeneration that might have been caused by EL.^16^, ^48^ During the Asian flu pandemic, the presence of influenza A virus was reported in the CSF sample of one patient.^45^ Various neuro‐psychiatric manifestations, including an altered mental state for more than 24 h, were also reported during the recent outbreak of H3N2 in 2017–2018.^45^, ^49^

The first human coronavirus was identified by Tyrrell and Bynoe in 1965 while working with human embryonic tracheal organ culture generated from the respiratory tract of an adult with a common cold.^50^, ^51^ Since then, seven different types and more than 30 different strains have been identified.^52^ Three of these, Severe Acute Respiratory Syndrome associated Coronavirus (SARS‐CoV), Middle East Respiratory Syndrome Coronavirus (MERS‐CoV), and SARS‐CoV2, showed high transmissibility and infectivity that resulted in the epidemics/pandemics.^50^, ^53^ Numerous cases of SARS‐CoV and MERS‐CoV infections have been associated with neurological illnesses, including neuropathy, delirium, and severe cerebrovascular disease.^54^ The recent outbreak of SARS‐CoV2 has been associated with a plethora of peripheral and central nervous system complications, including anosmia, hyposmia, cerebral edema, encephalitis, and long‐lasting neurological and psychiatric manifestations.^55^ As there are common neurological symptoms observed for human coronavirus and influenza virus (Figure 1), it is important to ask whether SARS‐CoV2 infection could lead to a gradual and significant neurodegeneration in long‐COVID. This will be helpful to enhance the preparedness in our public health system.

## NEUROLOGICAL COMPLICATIONS DURING AND AFTER COVID‐19 INFECTION

On analyzing the data collected during January–February 2020 from COVID‐19 special care centers in Wuhan, the neurologists reported that 36.4% of 214 patients exhibited neurologic manifestations.^56^ Several studies reported cognitive problems persisting in patients postrecovery.^57^, ^58^, ^59^ The mean time for the onset of these symptoms was 1.95 ± 1.65 months (mean ± SD), suggesting a gradual neuronal loss and altered adult‐neurogenesis postinfection.^58^ This is supported by various reports on the presence of viral particles in the CNS and, thereby, the morphological aberrations.^14^, ^47^, ^60^ Prevalent neurological concerns of COVID‐19 include chronic taste and smell disturbances, vertigo, myalgia, fatigue, attention‐related problems, and memory impairments. Four months after hospitalization, cognitive impairments, anxiety, sadness, and posttraumatic stress disorder were shown to be considerably higher in a study of 478 COVID‐19 survivors.^61^ Small fiber neuropathy, refractory status epilepticus, postinfection myelitis, encephalomyelitis, and dysautonomia have also been reported in long‐COVID.^62^, ^63^, ^64^, ^65^, ^66^ Long‐term cardiovascular sequelae in the aftermath of COVID‐19 infections heightens the chances of cerebrovascular disorders, and dysrhythmias.^67^

Viruses can attack the central nervous system by hematogenous or transneuronal routes via peripheral nerves, nucleus solitarius of the brain stem, spinal cord, and olfactory and trigeminal systems. While the direct entry of the virus through the olfactory system is debated, cytokine storm and microglial activation, along with haematogenic factors, could be the mechanisms by which the virus affects various body organs, including the brain.^68^, ^69^, ^70^ Barrier penetration of SARS‐CoV‐2 spike protein's S1 subunit suggests an effective endothelial cell crossing in the human BBB. Inflammatory cytokine‐mediated breaching can destabilize the BBB, thereby facilitating the entry of circulating viral particles in the circumventricular organs.^69^, ^71^, ^72^ The systemic inflammation spurred by the circulating virus triggers microglial activation. This could lead to enhanced glutamate and quinolinic acid release, which in turn alter *N*‐methyl‐d‐aspartate (NMDA) receptor expression, thereby causing memory deficits.^69^, ^73^, ^74^ SARS‐CoV2 infection also causes changes in the gut‐microbiome and reactivation of several commensal pathogens like Epstein‐Bar virus, which could potentially lead to the neuro‐psychiatric symptoms of long COVID.^71^ Symptoms such as encephalitis, cerebral edema, and the increased chances of stroke have been correlated largely with the cytokine storm and immune cell infiltration via breached brain vasculature and hypercoagulable form of the virus.^68^, ^75^ Prevalent symptoms of anosmia and hyposmia in COVID‐19 patients were explained by the expression pattern of molecular factors mediating the viral entry, the ACE2 receptors and TMPRSS2, in the supporting sustentacular cells in the olfactory epithelium.^13^, ^76^ However, whether the virus can enter the CNS via the olfactory tract is not clear, as there are conflicting findings on the presence of viral particles in the olfactory system.^14^, ^29^, ^77^

## NEUROTROPISM OF SARS‐COV‐2: CONFLICTING EVIDENCE

Influenza viruses and coronaviruses have the capability to invade the highly protected CNS. The olfactory mucosa, which is in continuous exposure to the environment, hosts supporting cells that express ACE2 receptors and the TMPRSS2, the molecular factors that facilitate the virus entry.^13^, ^76^ The cranial nerves, for example, the trigeminal and olfactory nerves, can act as a structural link between the exposed nasal cavity and the CNS via their projections. The other cranial nerves, such as the facial, glossopharyngeal, and vagal nerves innervating the respiratory tract and projecting to the brain, can also act as entry routes.^78^ In summary, the SARS‐CoV‐2 virus can potentially reach the CNS via olfactory, ocular, and gustatory pathways, as well as hematogenous pathways. The latter is comprised of the choroid plexus, BBB, blood–cerebrospinal fluid barrier (CSF), and enteric nervous system.^13^, ^27^, ^76^, ^79^, ^80^ Several in vitro and in vivo models have investigated the infectivity of neuronal cells. This includes animal model studies, as well as studies using cell culture and brain organoids.^81^, ^82^ The neuroinvasive potential of SARS‐CoV‐2 has also been investigated in mouse models by expressing the hACE2 receptor under the K18 promoter. These studies show increasing viral titers in the mouse brains following intranasal infection of SARS‐CoV‐2.^83^ In addition to the infection of neurons, the supporting glial cells in the brain are also potentially susceptible to infection and are also known to play an important role in the critical functioning of the brain and its immune response. Evidence for infection of glial cells by SARS‐CoV‐2 and its implications on brain function is accumulating in the literature. Glial cells are the primary inflammatory and immune cells. They play a critical role in properties like plasticity, neurogenesis, synaptic connections, etc., and thus are known to be important players in the process of neurodegeneration.^84^, ^85^, ^86^

An early study during the outbreak of COVID‐19 using autopsy samples found evidence of multiorgan viral invasion and viral RNA copies in brain tissue.^60^ Subsequent studies revealed the presence of viral nucleocapsid protein in the olfactory nerve, medulla, and forebrain of the infected patients.^87^, ^88^ Further research has reported key findings about the viral loads in different parts of the body, including respiratory/non‐respiratory organs.^89^ Presence of the virus has been confirmed in the olfactory nerve and nasal mucosa,^90^ olfactory bulb, brain stem,^91^, ^92^ amygdala, entorhinal cortex,^93^ and neural and capillary tissue of the frontal lobe.^94^ Several investigators have found viral RNA persistence of SARS‐CoV2 in CNS as late as 230 days postinfection from an autopsied brain of a patient, spurring the debate of potential neurotropism and viral survivability in neural tissue. SARS‐CoV‐2 RNA and protein were detected in the hypothalamus and cerebellum of patients as early as P38 (postinfection day 38) and in the cervical spinal cord and basal ganglia at P40 and P42, respectively.^89^ All these studies confirm the neuroinvasive nature of SARS‐CoV‐2. However, some recent studies did not find the SARS‐CoV‐2 virus remnants in the CNS.^29^, ^77^, ^95^ In contrast to direct neural infection, some studies present evidence that neuro‐inflammatory complications within the CNS from virus‐induced vascular injury are likely involved.^95^

The conflicting results on neurotropism, paired with the various possible entry routes of the virus, raise questions of neuronal versus nonneuronal routes of invasion. The idea of axonal entry of the virus has been tempered by technical limitations. For example, the findings of Meinhardt et al.^14^ on the presence of virus nucleoproteins between the olfactory nerve layer and OB were questioned, as it was not clear whether the virus was present in OSNs or in the ACE2‐expressing supporting cells that interweave among the OSN axons. Ultrastructural investigations using electron microscopy (EM) in autoptic human samples, as well as in animal models, did not help to support the concept of direct neuronal infections.^84^, ^96^, ^97^ MRI brain scans of more than 700 subjects pre‐ and postinfection have shown considerable reduction in the gray matter thickness of the orbitofrontal cortex and parahippocampal gyrus and brain volume. Proxies of tissue damage and neurodegenerative spread were identified in the limbic and olfactory cortical system.^47^ Irrespective of the entry routes, all these studies suggest that dysfunction of various cell types occurs in the brain, collectively leading to CNS abnormalities and neurological problems.

## VIRUS‐INDUCED NEURODEGENERATION: FOCUS ON PARKINSON'S DISEASE

Neurodegeneration is the progressive loss of neurons in the CNS and peripheral nervous system. Neurodegenerative diseases can be caused by genetic factors and predispositions, as well as multiple environmental and physiological events that occur throughout a lifetime, including viral infections. Global estimates in 2019 showed over 8.5 million individuals with PD, and it is estimated that the number of individuals affected by PD will be 13 million by 2040 (WHO data, August 2023). In fact, only 2%–10% of PD cases are linked to genetic susceptibility or familial history.^98^, ^99^ Even though there have been debates regarding the spread of pathology in PD, studies have shown that the earliest PD‐related lesions appear in the olfactory bulb, the dorsal vagal nucleus, and the peripheral autonomic nervous system.^100^, ^101^ There are also anatomical connections between the olfactory bulb and the brain stem.^102^ Experimental studies on humans and animal models have suggested that transneuronal propagation of misfolded alpha‐synuclein is involved in the progression of PD.^103^ The enteric nervous system and brain are closely connected via axons of the efferent fibers of the dorsal nucleus of the vagus nerve. Experimental studies using animal models of PD suggest that the potential mechanisms for disease propagation could involve both retro‐grade (gut‐to‐brain) or anterograde (brain‐to‐gut) axonal transport.^104^

The link between viral infections and the development of neurodegenerative diseases, primarily Parkinson's disease, has been historically investigated, as outlined in Figure 1. The first case of sporadic PD that may have been linked to viral encephalitis dates back to the 1918 influenza pandemic.^105^ The correlation between events of viral infections such as Japanese encephalitis B virus, West Nile virus, HIV, and Influenza and the subsequent development of Parkinson's disease have been explored extensively and documented in the literature.^98^

One of the earliest documented case studies of a patient who developed sporadic PD post‐COVID was published in October 2020. The patient did not have any known genetic predisposing factors for PD and was diagnosed with PD a few days after recovering from COVID.^106^ Following this study, a number of cases have been reported, building reasonable evidence for a potential link between COVID‐19 and sporadic PD.^92^, ^107^ Studies have been conducted in PD patients to develop an understanding of the possible association with viral infections. In a study by Fazzini et al.,^108^ it was found that the CSF of PD patients showed a higher response to certain coronavirus antigens compared to age‐matched controls. Viruses that infect the brain lead to encephalopathies and can have the potential to trigger neurodegeneration and the development of PD through multiple pathways.^98^ COVID‐19 infection can be considered as a potential “environmental factor” in triggering the development of PD in individuals carrying genetic susceptibility factors like the GBA and PRKN gene variants.^109^

Some of the potential links between the SARS‐CoV‐2 infection and the development of PD arise from the pathways common to both diseases. The prodromal developmental stages of PD coincide with some of the typical SARS‐CoV‐2 infection symptoms and pathology.^110^ This is primarily seen in pathophysiology concerning the olfactory and gastrointestinal systems. According to Braak's hypothesis, sporadic PD development can originate from a pathogen entering the body via the nasal cavity or gut and can subsequently migrate to the brain, facilitating Lewy body formation.^111^ Hyposmia and GI dysfunction are commonly observed as early symptoms of PD and often precede the formal PD diagnosis.^99^ Aggregation of alpha‐synuclein (ASN) and formation of Lewy bodies are the characteristic pathophysiological features of PD. Upregulation of ASN has been observed in cases of viral encephalitis. The excess ASN production as a result of immune responses to infections can facilitate the accumulation and aggregation and hence could result in augmentation of neurodegeneration.^112^

Although clear causal evidence has not yet been established, emerging data are accumulating that suggest possible mechanistic links between COVID‐19 and PD. In an in vitro study using SH‐SY5Y cells, the N‐protein of the SARS‐CoV‐2 virus was found to speed up the aggregation of ASN, thus facilitating amyloid fibril formation.^113^ Another study found that the ACE2 receptor was expressed in hPSC‐derived dopaminergic cells, thus making them susceptible to viral infection and hence degeneration.^114^ In some animal studies, the viral antigen was detected in the subthalamic nucleus and substantia nigra (SN). Pathologies like neuronal loss and gliosis occurred in these regions after infection of the mice with the coronavirus MHV‐A59.^115^ Infecting macaques with SARS‐CoV‐2 also showed aggregates of ASN after infection, in combination with other neurological pathologies.^116^, ^117^ Previous work indicates that a neuroinflammatory response followed the activation of toll‐like receptors (TLRS), especially TLR4. Analysis of the transcriptomic data from postmortem samples of PD patients' brains reveals that there are notably enhanced levels of TLR‐4 expression in the substantia nigra and putamen. It has also been found that the TLR‐4 levels are enhanced in COVID‐19 patients, in support of the relationship between COVID‐19 and PD.^118^ The neurodegeneration due to SARS‐CoV‐2 could be triggered by the binding of SARS‐CoV‐2 spike protein to TLR4, affecting the dopaminergic neurons in the nigrostriatal system. Subsequently, the activation of intracellular signaling pathways involving MYD‐88 (myeloid differentiating primary response gene 88), coupled with the upregulation of inflammatory signaling molecules, can lead to NF‐kB‐induced release of cytokines, reactive oxygen species (ROS), and reactive nitrogen species (RNS). All these factors eventually contribute to neurodegeneration.^119^

A number of studies have been published over the last 2 years that have evaluated the impact of SARS‐CoV‐2 on PD patients. Overall, conclusions indicate that COVID‐19 infection can accelerate disease progression.^120^ An online study conducted by Fox Insight Foundation concluded that, among the COVID‐19‐infected PD patients, 55% showed severity in their existing motor symptoms, and 18% reported developing new motor symptoms. These patients also showed the occurrence or worsening of non‐motor symptoms in a relatively high proportion, including mood symptoms (71%), sleep disruptions (62%), cognitive problems (49%), and dysautonomia (38%).^121^ A retrospective longitudinal study was conducted in Egypt where the progression of the motor symptoms was measured using the MDS‐UPDRS‐Ⅲ scale (Movement Disorder Society‐Unified Parkinson Disease Rating Scale‐Ⅲ) during the onset of COVID‐19 from December 2019 to April 2020. A marked deterioration of the motor symptoms and higher motor disease progression was observed.^122^ The PD‐related mortality rate also increased steeply during COVID‐19 progression. A retrospective analysis of 70 PD inpatients in New York found that PD patients with COVID‐19 infection had a greater mortality rate (35.8%) as compared to those not infected with COVID‐19 (5.9%).^108^ While several explanations, such as altered dopamine metabolism, reduced dopaminergic drug response, and modified transport of dopaminergic drugs through the BBB, can be found for the worsening of PD, one cannot exclude other general reasons such as changes in routine life and exercise, stress, anxiety, prolonged immobility, and social isolation.^123^

Clinical reports investigating various aspects of brain pathophysiology provide the potential link between Parkinson's disease and viral infections, including SARS‐CoV‐2. However, to move toward the causality and circuit mechanisms, a multipronged experimental approach using animal models is needed.^124^, ^125^, ^126^, ^127^, ^128^, ^129^ Different animal models that can be used to predict adverse effects of long‐COVID.^82^, ^127^, ^130^ Long‐term experiments in which SARS‐CoV‐2 infection is introduced into PD animal models or in which parkinsonism is induced in COVID‐19‐infected animals would provide answers to many unsolved questions.

## OLFACTORY DYSFUNCTIONS: A COMMON FACTOR BETWEEN LONG COVID AND PARKINSON'S DISEASE

Long COVID refers to the health problems in the aftermath of active COVID‐19 infection. These include long‐lasting respiratory, metabolic, cardiovascular, and neurological symptoms. Since olfactory dysfunction occurs in both PD and COVID‐19, such dysfunction could reflect a common link between the two. Olfactory disorders, including anosmia, hyposmia, and parosmia, were classified as prevalent symptoms of chronic COVID‐19, and the presence of the viral particles was detected in the olfactory bulb of COVID‐19 patients.^96^ In the past 2 years, there have been numerous reports of long‐lasting anosmia and altered sense of smell, or parosmia and phantosmia, in otherwise recovered COVID‐19‐infected subjects.^131^, ^132^ Anosmia and hyposmia can be explained by the loss of olfactory sensory neurons (OSNs) in the olfactory mucosa and/or reduction in the sensitivity due to aftereffects of SARS‐CoV2 infection in supporting cells. Parosmia may result from the lack of a full complement of afferent information from the limited number of multiple receptors caused by damage or from unusual rewiring of newly formed OSNs in the olfactory bulb (OB) or both.^133^ Parosmic conditions are frequently associated with a partial recovery of the sense of smell that affects the quality of life, food choices, and mental states of the patients.^134^ Self‐reported ill‐effects of olfactory dysfunctions in recovered COVID‐19 subjects include adversities in daily life, including altered dietary habits, appetite loss, a feeling of dissociation, detachment, and despair.^131^, ^132^, ^134^ In 40.3% of the long‐COVID cases, parosmia prevailed for nearly 2.5 months in a cohort of 613 COVID‐19 recovered patients.^135^ Partial recovery of anosmia has been documented in a young patient 15 months post‐onset, with severe parosmia.^136^

**Figure 2:**
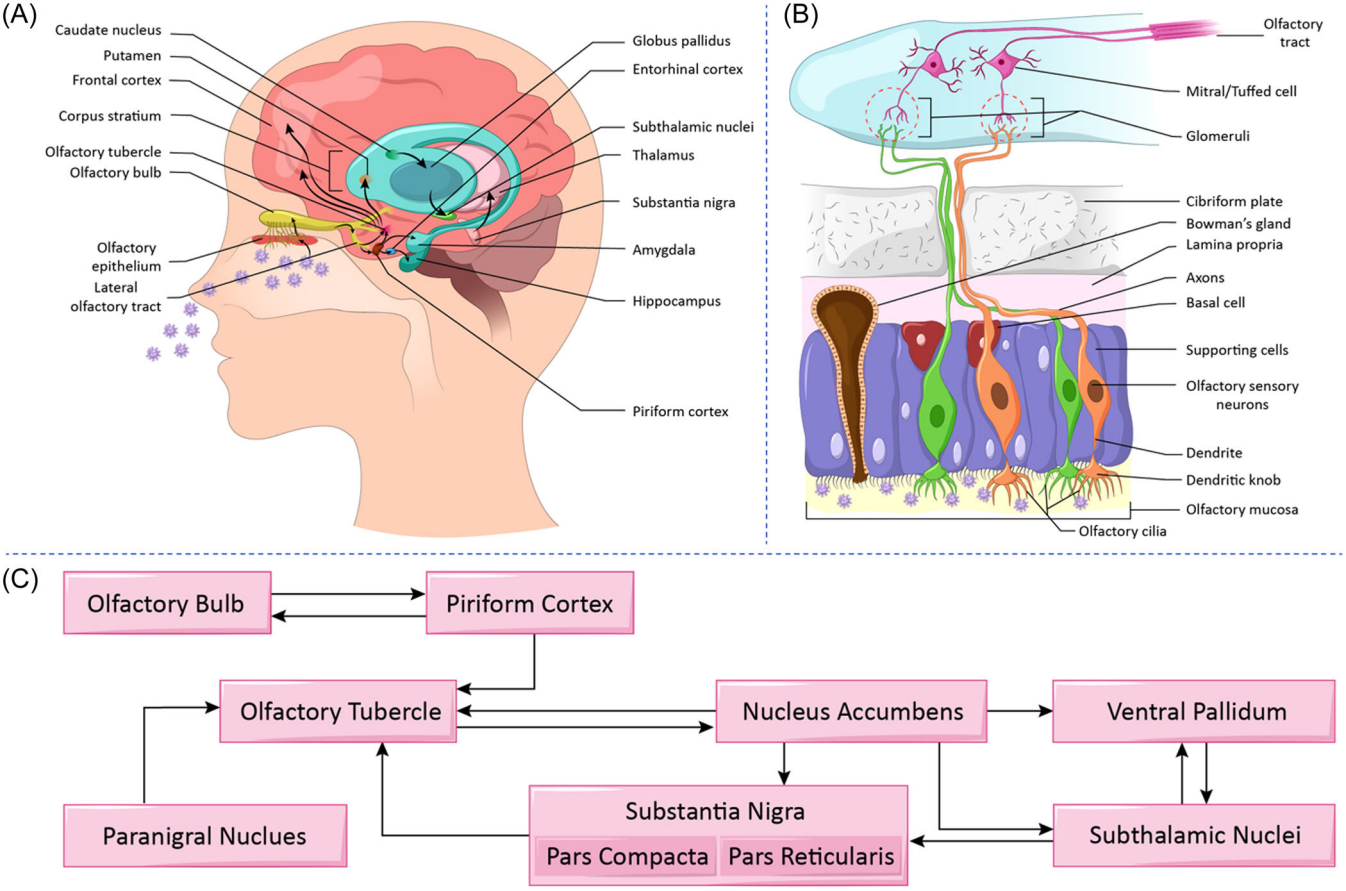
Understanding potential pathways underlying long‐term pathological consequences of coronavirus disease 2019 (COVID‐19) and incidence of Parkinson's Disease. (A) Schematic representation of potential transneuronal viral transmission routes. This depicts anatomical connections of olfactory cortical centers with mesocorticolimbic and nigro‐striatal dopaminergic pathways involved in Parkinson's Disease pathology. (B) Schematic representation of olfactory pathway as a potential route for the central nervous system (CNS) invasion of severe acute respiratory syndrome coronavirus 2 (SARS‐CoV‐2). Olfactory system is highly vulnerable to external infectious agents due to its anatomical position. Bowman's gland secretion forms the mucosal lining of the olfactory epithelium, wherein viral particles get captured and largely infiltrate supporting cells, eventually affecting adjacent olfactory sensory neurons. The continuous connectivity between olfactory sensory neurons with M/T cells of the olfactory bulb could facilitate CNS invasion and transmission of the virus to different parts of the brain. (C) The diverse connectivity of olfactory cortical centers, i.e., piriform cortex and olfactory tubercle, with that of substantia nigra, subthalamic nucleus, and striatum that are involved in Parkinson's disease pathology.^68^, ^139^, ^140^, ^141^

Olfactory impairment appears to be one of the most reliable biomarkers of a number of neurodegenerative disorders, including AD, PD, Multiple System Atrophy (MSA), and some other synucleopathies.^137^ Strikingly, hyposmia and anosmia are the symptoms often seen much earlier than the development of any motor dysfunctions in PD patients.^138^ Olfactory dysfunctions are also seen in post‐COVID subjects even 1 year after recovery, indicating persistent long‐term damage to the olfactory system. Deficiency observed in olfactory matching, where the working memory is involved, implies long‐term cognitive problems that could exist in long‐COVID.^57^ The anatomical position of the OB, receiving the inputs from the external nasal cavity, as well as numerous projections that the OB has with higher brain areas such as the cortex, striatum, and amygdala, make the OB a potential hub for the virus to spread into other regions of the brain that are relevant in neurodegeneration (Figure 2).^102^

As the olfactory system is directly exposed to the external environment, it is vulnerable to various toxic insults and other risk factors for neurodegenerative diseases. The olfactory vector hypothesis states that various toxins and other risk factors could directly access the brain via this pathway, which triggers various disorders such as Alzheimer's and Parkinson's disease.^140^ Olfactory dysfunctions reported at the early stage of PD include deficits in odor identification, discrimination, threshold detection, and olfactory memory.^142^ The early pathological deposition of alpha‐synuclein in primary and secondary olfactory centers and other factors, such as hippocampal dysfunction, along with cholinergic and dopaminergic denervation, contribute to the PD pathology observed in the OB.^143^ The anterior olfactory nucleus could play an important role in the spread of pathology because of its connectivity with higher brain areas.^144^ Additionally, the neuromodulatory serotonergic and noradrenergic pathways to the OB are also involved in the alpha‐synuclein spread during the disease progression. Significant olfactory loss and progressive reduction of cortical thickness and basal ganglia volume due to the anterograde spread were observed in a longitudinal study in PD patients.^145^

## CONCLUSIONS

Given the facts recorded in the history of viral epidemics/pandemics, along with the clinical reports of neurological complications available thus far from long‐COVID subjects, lead one to consider a potential link between SARS‐CoV‐2 infection and neurodegeneration. As discussed throughout this article, evidence is accumulating on the link between COVID‐19, its long‐term neurological impact, and neurodegeneration. Medical reports and documented cases of the long‐term consequences of acute COVID, long‐COVID, and sporadic PD cases post‐COVID‐19 have been published. However, definitive data on the overlap between COVID‐19, olfactory dysfunctions, and development of PD are lacking. The focus should be on patients with long COVID, diagnosing olfactory dysfunctions, and tracking these patients to judge whether they are developing strong neurodegenerative symptoms. These observations need to be supported by long‐term experiments using animal models of COVID‐19 and neurodegenerative disorders. Developing credible animal models of COVID‐19 has been a challenge, and several models are used now to address various questions related to long‐COVID. As olfaction is commonly affected in long COVID and neurodegenerative disorders, the experimental strategies can be built on by making use of olfactory systems' functions. To precisely define the affected neural circuits and to understand the causes of COVID‐19 pathologies, multipronged experiments employing animal models are sorely needed. Such experiments will help to connect the dots between long COVID, olfactory dysfunction, and the development of PD and a number of other neurodegenerative diseases.

## AUTHOR CONTRIBUTIONS

Sanyukta Pandey, Vibha Bapat, Jancy Nixon Abraham, and Nixon M. Abraham wrote the manuscript. Jancy Nixon Abraham, and Nixon M. Abraham edited the manuscript.

## CONFLICT OF INTEREST STATEMENT

The authors declare no conflict of interest.

## ETHICS STATEMENT

None declared.

## Data Availability

We have mostly used Google Scholar and PubMed to search for the articles. As we aimed to provide a narrative overview on the topic of Review, we are summarizing the selected and relevant literature.
